# Epidemiology of Traumatic Head Injuries in Sweden during 2008–2022

**DOI:** 10.1159/ned/adwag021

**Published:** 2026-10-10

**Authors:** Jia-Cheng Xu, Zhou Zhou, Christer Ahlström, Svein Kleiven

**Affiliations:** a Division of Neuronic Engineering, KTH Royal Institute of Technology, Huddinge, Stockholm, Sweden; b Swedish National Road and Transport Research Institute (VTI), Linköping, Sweden; c Department of Biomedical Engineering, Linköping University, Linköping, Sweden

**Keywords:** Traumatic head injury, Traumatic brain injury, Epidemiology, Ageing population, Vision Zero

## Abstract

**Background:**

Traumatic head injuries, including fractures and traumatic brain injuries (TBIs), are primarily caused by falls and road traffic accidents (RTAs). Since the introduction of the Vision Zero policy in Sweden in 1997, falls have overtaken RTAs as the main cause of traumatic head injuries. This study aimed to estimate national injury rates of fractures and TBIs in both inpatient and outpatient specialised care in Sweden from 2008 to 2022.

**Methods:**

Data from the Swedish National Patient Register from 2008 to 2022 were stratified by external cause, sex, age, and injury (fractures and TBIs). Changes over time were analysed using generalised linear models with either linear or quadratic functions and with or without a first-order autoregressive correlation structure. Holm-Bonferroni-adjusted *p* values were applied to account for multiple comparisons.

**Results:**

Rates of traumatic head injuries, TBIs, and fractures declined for both sexes, with less pronounced decrease among women. Falls remain the primary cause of head injury with a minor decrease for men and no reduction for women from 2008 to 2022. Falls accounted for 62–67% of traumatic head injuries in women and 46–57% in men throughout the study period. Transport-related injuries remained below 20% for both sexes. While concussions and fractures declined overall, fracture rates among women remained stable. Subdural and subarachnoid haemorrhages (SDH and SAH) nearly doubled and tripled in incidence, respectively, between 2008 and 2022. Head injury rates were highest among individuals aged 85+, with SDH and SAH trends closely mirroring demographic shifts.

**Conclusions:**

Including outpatient care data improves the accuracy of head injury estimates, especially for concussions, which are often underreported in inpatient-only studies. The rising incidence of SDH and SAH, along with an increase in head injuries among age 85+, highlights the need for targeted prevention strategies towards the oldest-old population. While Vision Zero has successfully reduced transport-related injuries, urgent efforts are needed to address fall-related head injuries.

## Introduction

The main purposes of utilising healthcare data to conduct injury epidemiology studies are to evaluate the effectiveness of previous and ongoing efforts and to plan and prioritise future efforts. These efforts aim to prevent injuries, deaths, long-term impairments, and societal costs. The estimated global incidence of traumatic brain injuries (TBIs) considering all causes is around 69 million new cases per year, with road traffic accidents (RTAs) accounting for a greater proportion of head injuries in low- and middle-income countries [1]. In Sweden, traumatic injuries caused by external forces, such as in RTAs, have been targeted through the “Vision Zero” policy. Vision Zero was adopted by the Swedish parliament in 1997, with the goal that no one should be killed or get seriously injured in an RTA. Since then, it has been gradually implemented across Europe and is being adopted on other continents. However, the effect is mostly seen in high-income countries (HICs), which in the case of head injuries can explain why TBI cases in low- and middle-income countries are still primarily caused by RTAs. Recent shifts in TBI incidences across Europe have revealed that falls have surpassed RTAs as the leading cause of TBIs in HICs [2–5].

National-level epidemiological studies on head injuries remain scarce, raising concerns about the quality and completeness of injury data. In Europe, the living systematic review by Brazinova and colleagues [4] provides up-to-date incidence rates of TBIs, based on scientific publications using nationwide or region-level data. The review identified only 13 nationwide studies, highlighting a lack of both high-quality data and comprehensive publications across Europe. Most nationwide studies included in the review were based on inpatient care (hospital admission) data. While Majdan and colleagues [3] include data on TBI-induced hospital discharges from European countries, the study is limited to 2012 and reports only aggregated TBI incidence rates. Inpatient care data likely underestimate the true incidence of TBIs, especially mild TBI (mTBI, or concussion) [6–9]. This limitation makes inpatient data insufficient for accurately estimating the overall burden of traumatic head injuries. The reliance on inpatient data is especially concerning given the long-term reduction in hospital bed capacity in HICs, with Sweden having one of the lowest per capita rates for several decades [10].

In Sweden, only two nationwide studies investigated the incidence and causes of traumatic head injuries (including both fractures and TBIs). A shift towards falls becoming a more common cause of traumatic head injuries than RTAs was observed in Sweden more than 25 years ago by our previous studies [11, 12]. These investigations reported injury rates from inpatient care during 1987–2000 to 1987–2010, respectively. Both studies showed that overall head injury rates were decreasing but increasing for ages 85+, that men had nearly twice the injury rates compared to women, that concussion rates were declining, and that traumatic haemorrhages were increasing. However, both investigations were limited to inpatient care data only. There is a need for comprehensive and updated nationwide statistics on head injuries to guide future prevention measures, considering the increasing and worrying trends observed in the Swedish data from 1987 to 2010.

To the authors’ best knowledge, no previous nationwide epidemiological studies have provided traumatic head injury rates using data covering all specialised care visits (defined as both inpatient and outpatient care), without aggregating fractures and specific TBIs (i.e., failing to present the incidence rates for each specific TBI code). The main objective of this study is therefore to fill this gap by providing a comprehensive national overview of traumatic head injuries in Sweden, based on data from all specialised care visits from 2008 to 2022, and reporting injury rates for both TBIs and fractures.

## Methods

Both inpatient and outpatient care data were extracted from the Swedish National Patient Register (NPR) by a statistician at the National Board of Health and Welfare. NPR is a high-quality source commonly used in Swedish epidemiological research. It does not include primary care data, which were excluded from this study due to inconsistent quality and decentralised access.

### Data Acquisition and Stratification

The delivered dataset was anonymised and contained no personal information, and hence, no ethical approval was required. The injury dataset included only primary diagnoses to capture first-time injuries, representing the main reason for the patient’s healthcare contact. To avoid counting repeated visits related to long-term consequences of the initial injury, a 2-year washout period was applied. This approach aimed to minimise overestimation and better reflect newly sustained injuries.

The data were provided as absolute numbers of injuries and injury rates per 100,000 population, annually from 2008 to 2022. Although data collection in outpatient care began in 2001, only data from 2008 onwards are of sufficient high quality for epidemiological purposes. During this period, the Swedish population increased from 4,603,710 to 5,298,324 among men and from 4,652,637 to 5,223,232 among women.

The stratified variables are defined according to ICD-10. To clarify that the definition of TBIs does not include fractures, “traumatic brain injury” and “intracranial injury” are used interchangeably with the latter reflecting the ICD-10 terminology. As such, the injury types and their causes were stratified as:

- Traumatic head injuries: fractures of cranial and facial bones (S02) + intracranial injuries (S06).
- TBI: intracranial injuries (S06).
- Fractures of cranial and facial bones (S02).
- External causes V01–Y98 (aggregated codes per category): falls (W00–W19), transport (V00–V99), violence (X60–Y09, Y35–Y36). Remaining codes in V01–Y98 grouped as “other,” i.e., W20–X59, Y10–Y34, Y40–Y98, and “missing,” i.e., injuries with no coded external cause.
- Specific TBIs: S06.0–S06.9.

### Statistical Analysis

Descriptive statistics were used to analyse the raw longitudinal data, providing an overview of variability of head injury across the study period. Changes over time in injury rates across different sex-category strata – defined by sex in combination with either external causes, age groups, or specific injury types – were assessed using generalised linear models (GLMs). For each group, Poisson and negative binomial GLMs were evaluated, using annual injury counts as the outcome and the logarithm of the corresponding annual population as an offset. This model approach accounts for annual injury rates and differences in population sizes. One joint GLM was fitted per group to include all sex-category strata as fixed effects under the same distribution and temporal model properties. Interactions between the sex-category strata and year were included to estimate separate baseline rates and changes over time for every stratum. To allow for both linear and nonlinear longitudinal trends, we evaluated linear, quadratic, and spline functions of year. Lastly, due to potential correlation between annual rates and an expected decay in correlation over time, the GLMs were also evaluated with and without a first-order autoregressive structure. Collectively, a small-sample corrected Akaike information criterion (denoted as AIC_C_) was used to inform the choice of the final GLM model properties mentioned above for each group. Due to multiple comparisons across the sex-category strata, the Holm-Bonferroni correction was applied within each group to control the family-wise error rate. The significance level was set to *p* < 0.05. All statistical analyses were performed in R version 4.5.0.

## Results

The following subsections present injury rates per 100,000 for each group containing various sex-category strata. Figure 1a–c covers sex-cause strata for different groups of traumatic head injuries. Figure 2 presents mean traumatic head injury rate trends for different age groups. Figure 3 shows sex-age group strata for all traumatic head injuries. Figure 4a–c stratifies traumatic head injuries to present sex-injury strata for both specific TBIs and aggregated fractures.

**Fig. 1.**
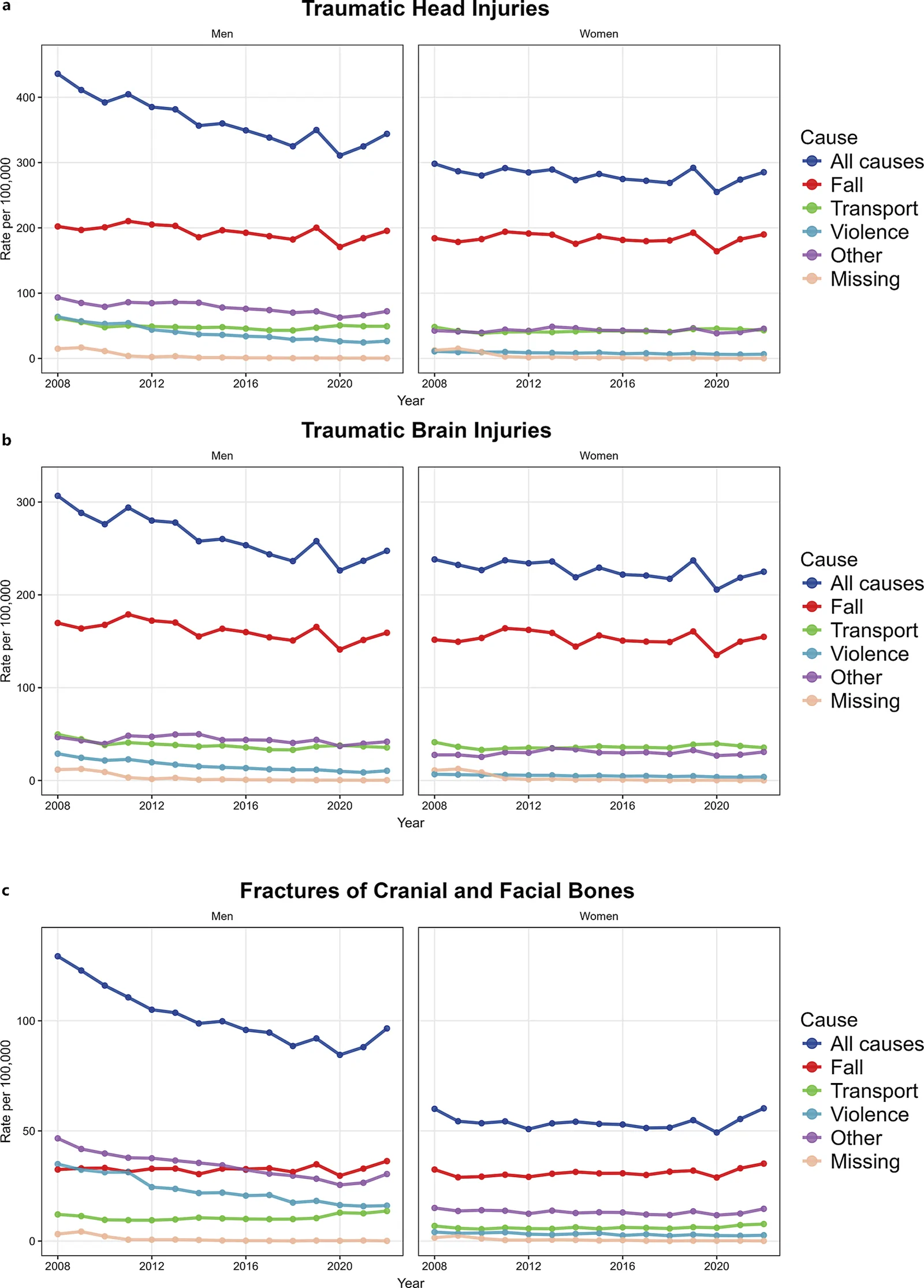
Head injury rates from 2008 to 2022 stratified by sex and external cause (fall, transport, violence, others, missing), divided into traumatic head injuries (**a**), only TBIs (**b**), and only fractures of cranial and facial bones (**c**).

**Fig. 2.**
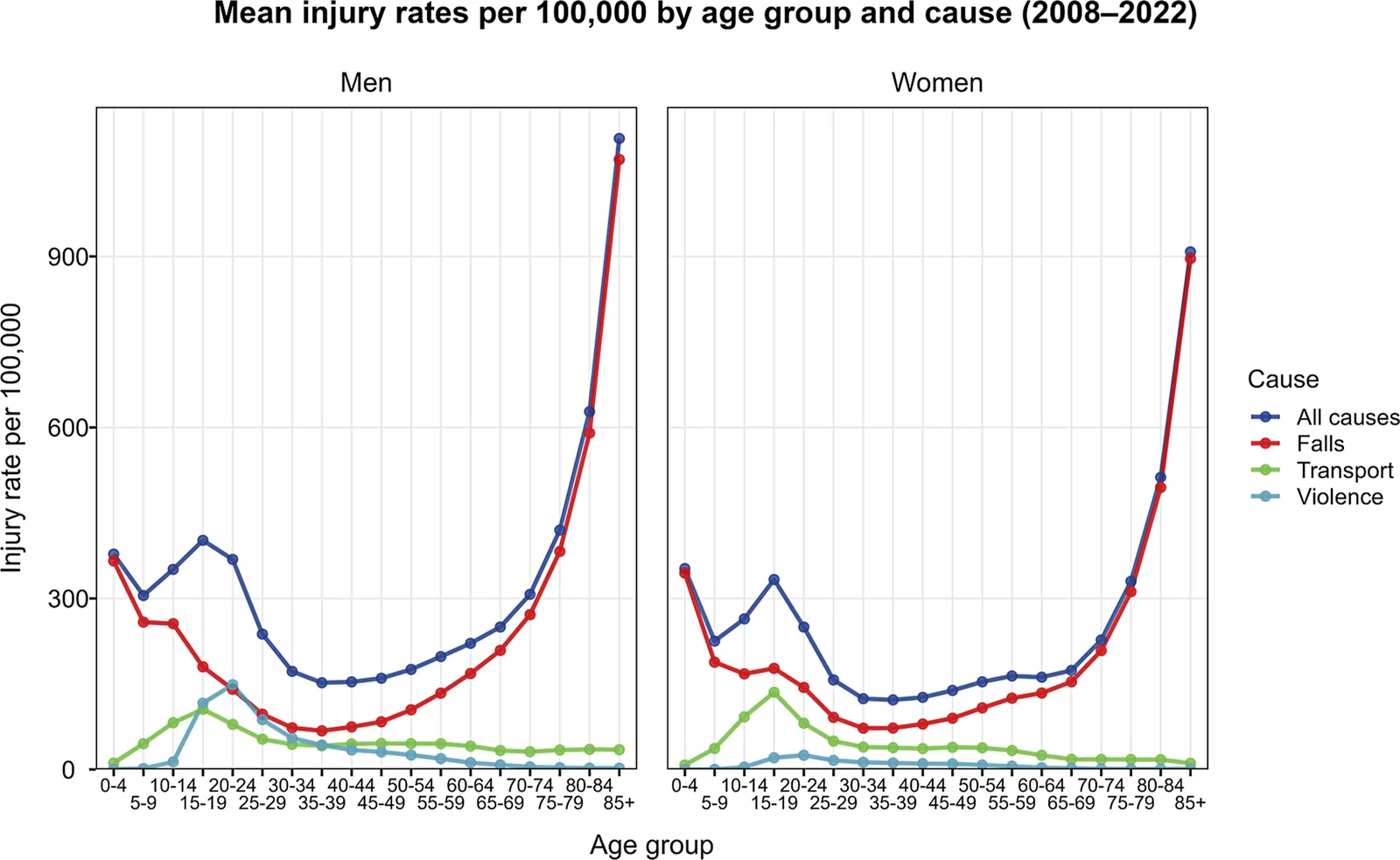
Mean head injury rates stratified by the main external causes over the period 2008–2022. Other and missing cases were omitted since it fails to describe the accident type.

**Fig. 3.**
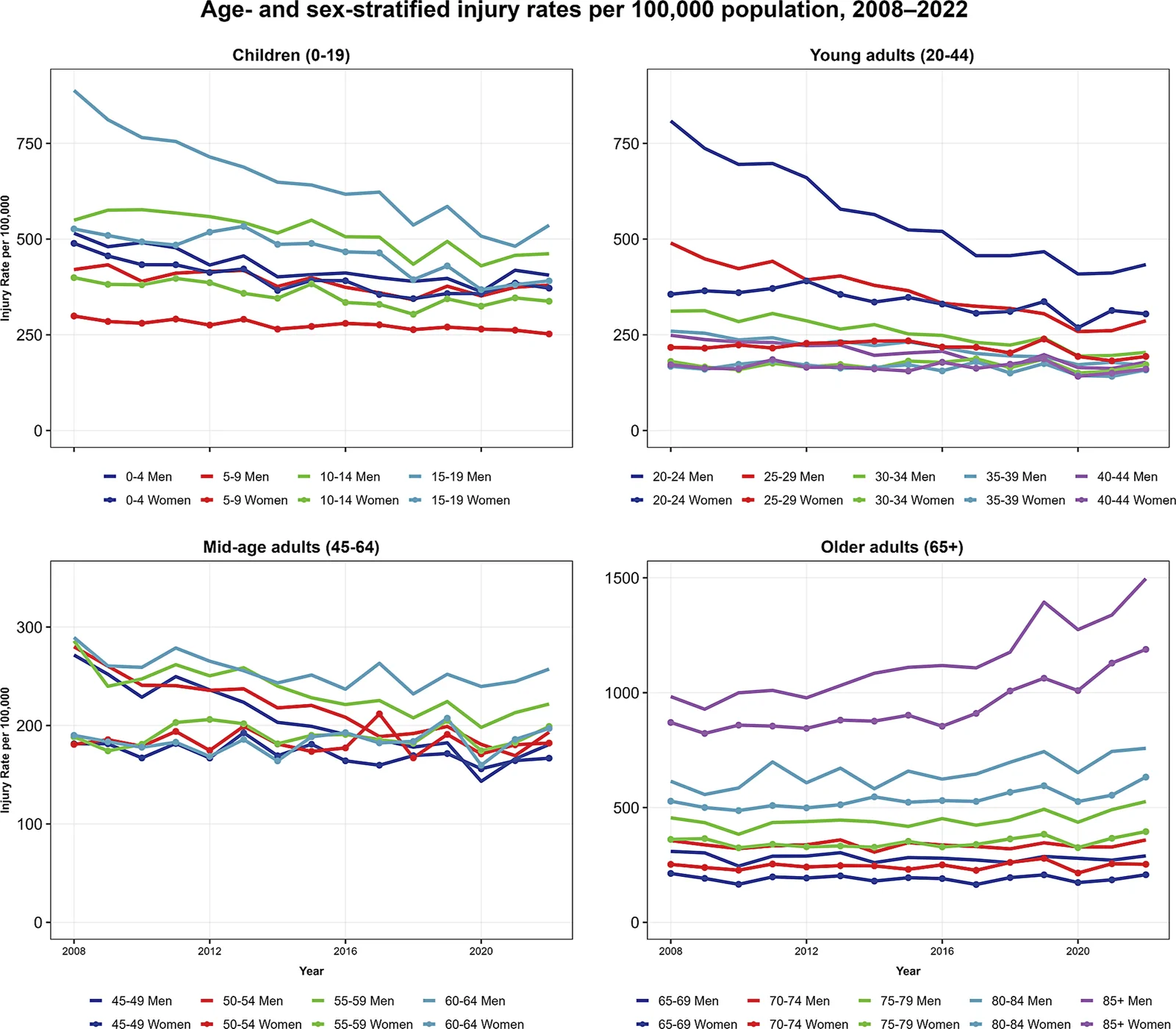
Annual age and sex differences in head injury rates from 2008 to 2022.

**Fig. 4.**
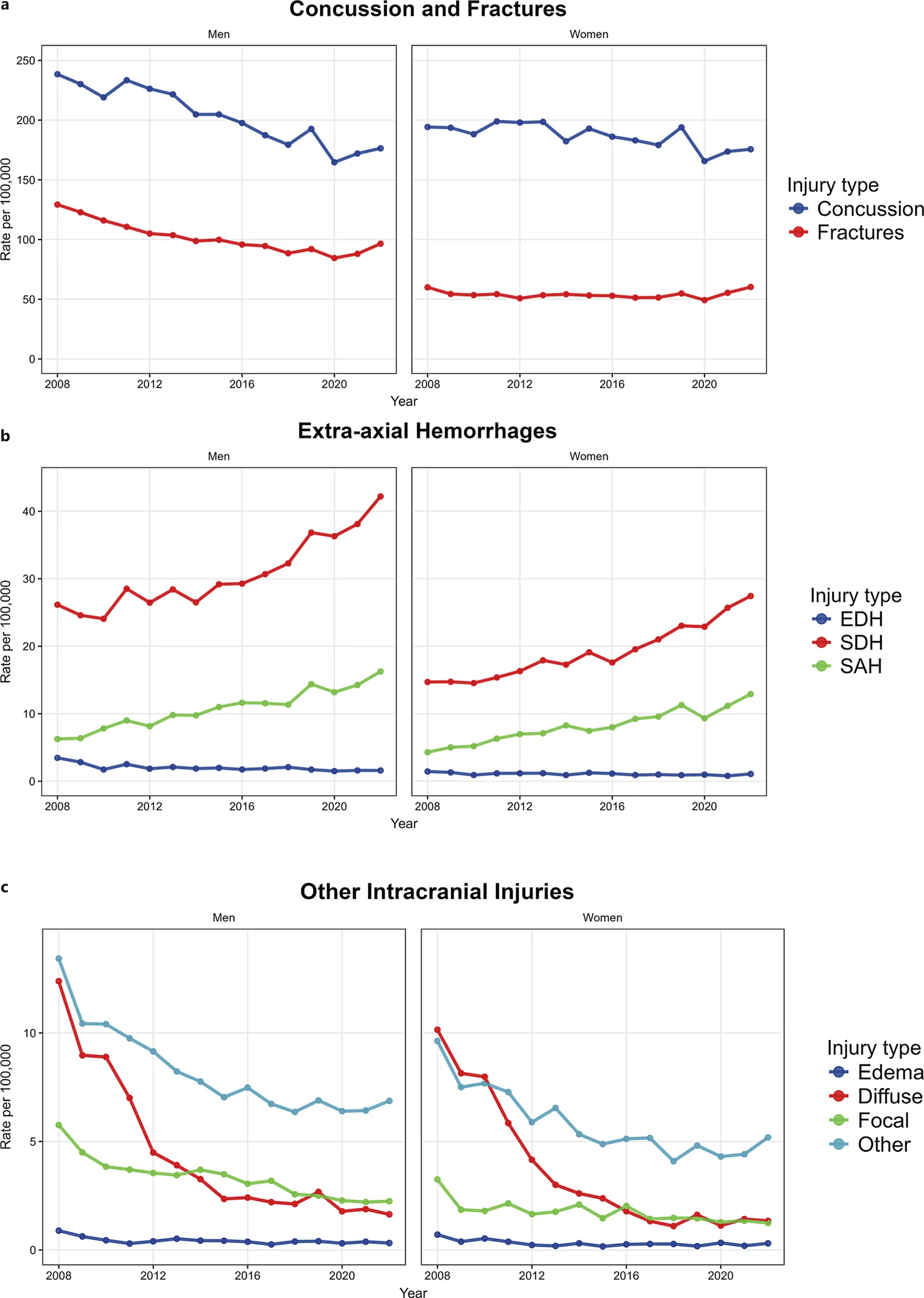
Specific types of traumatic head injuries according to ICD-10, from 2008 to 2022: concussion and fractures (**a**), epidural haemorrhage (EDH), subdural haemorrhage (SDH), subarachnoid haemorrhage (SAH) (**b**), other intracranial injuries defined by ICD-10 (other constitutes S06.7–S06.9) (**c**).

For each group (either subfigure or figure), the final statistical model candidates are shown in Table 1, where the lowest AIC_c_ informed the selection of the final model. The Holm-Bonferroni-adjusted *p* values are reported at a significance level of 5%.

**Table 1.** Selected GLMs for each group containing sex-category strata

Groups with sex-category strata	GLM
Figure 1a	Quadratic Poisson with AR1
Figure 1b	Quadratic Poisson with AR1
Figure 1c	Quadratic Poisson with AR1
Figure 3	Linear negative binomial with no AR1
Figure 4a	Quadratic negative binomial with no AR1
Figure 4b	Linear negative binomial with no AR1
Figure 4c	Quadratic negative binomial with no AR1

AR1, first-order autoregressive; GLM, generalised linear model.

From 2008 to 2022, head injury rates decreased from 436 to 293 per 100,000 among men and from 298 to 245 per 100,000 among women. This corresponds to a reduction of 33% for men and 18% for women.

### Head Injury Rates by Sex and External Cause

When examining the causes of head injuries from 2008 to 2022, falls consistently emerged as the primary cause. Fall-related head injuries were more common among women than men. Table 2 shows that falls accounted for 46–57% of head injury cases among men and 62–67% among women throughout the entire study period. The proportion of fall-related injuries increased over time, with higher percentages observed in later years. As shown in Figure 1a, the rates of fall-related head injuries in men were 202 per 100,000 in 2008 and 195 per 100,000 in 2022 (a 3% not statistically significant reduction, *p* = 0.89). Among women, the rates were 184 and 190 per 100,000 in those same years, a difference that was not significant (*p* = 1.00). Transport-induced head injuries occurred in similar proportions between the sexes, accounting for 12–16% of head injury cases among men and 14–18% among women. Changes in transport-related injury rates were only statistically significant for men from 2008 to 2022, but not for women. Among men, transport-related injuries decreased from 62 to 49 per 100,000 (significant, *p* = 0.02), while among women, the rates decreased from 48 to 43 per 100,000 (not significant, *p* = 1.00). Other significant changes from 2008 to 2022 were found among men, including a 58% decrease in violence-related injuries and a 23% decrease for injuries classified as other (both *p* < 0.0001). Additionally, injuries with missing cause information decreased significantly by 97% from 2008 to 2022 (*p* < 0.001). For women, the only significant changes were a 41% reduction in violence-related injuries and a 98% decrease in missing cases from 2008 to 2022 (both *p* < 0.0001). The significant reduction in the missing category occurred predominantly from 2008 to 2011 for both sexes. For the rest of the study period, from 2011 to 2022, this category contributed minimally to the total, between 0.2 and 3.9% for men and 0.2–2.9% for women, with a decreasing trend over time for both sexes. Violence-induced head injuries were more common among men than women, accounting for 8–15% of injury cases among men and 2–4% among women.

**Table 2.** Variation of proportions (denoted as mean, with range in parentheses) of annual injury rates by external cause, 2008–2022

​	Fall	Transport	Violence	Other	Missing
A. Traumatic head injuries					
Men	53.5% (46.4–57.2%)	13.5% (12.2–16.3%)	10.6% (7.6–14.6%)	21.4% (20.1–23.9%)	1.0% (0.1–4.1%)
Women	65.5% (61.7–67.2%)	15.2% (13.7–17.9%)	2.9% (2.2–3.7%)	15.3% (14.1–17.0%)	1.2% (0.1–5.2%)
B. TBIs					
Men	61.6% (55.3–64.3%)	14.6% (13.6–16.7%)	6.0% (3.7–9.4%)	16.7% (14.3–19.3%)	1.1% (0.1–4.3%)
Women	67.4% (63.7–69.3%)	16% (14.5–19.3%)	2.2% (1.6–2.9%)	13.1% (11.2–15.4%)	1.2% (0.1–5.4%)
C. Fractures of cranial and facial bones					
Men	32.5% (25.1–37.9%)	10.8% (8.3–15.2%)	22.4% (16.7–28.3%)	33.5% (30.1–36.1%)	0.8% (0.1–3.5%)
Women	57.3% (53.2–61.2%)	11.4% (10.1–13.1%)	5.8% (4.3–7.3%)	24.4% (22.5–26.2%)	1.1% (0.1–4.5%)

### TBI Rates by Sex and External Cause

Figure 1b and Table 2b show that TBI rates decreased from 307 to 247 per 100,000 among men and from 238 to 225 per 100,000 among women. Overall among men, statistically significant reductions were observed for all causes (*p* < 0.0001) except for falls and “other” (*p* = 0.47 and *p* = 0.18). Overall among women, only violence and missing categories showed statistically significant reductions (*p* < 0.0001 and *p* < 0.001). Falls are the most common cause of TBIs, accounting for 55–64% of cases among men and 64–69% among women. In detail, among men, fall-related TBIs decreased slightly but not significantly by ca. 6% (*p* = 0.47), and no significant change was observed among women as well (*p* = 1.00). Transport-related TBIs comprised 14–17% among men and 15–19% among women. A significant 28% decrease in transport-related TBIs was observed among men, from 50 to 36 per 100,000 between 2008 and 2022 (*p* < 0.001). Among women, the reduction of transport-related TBIs from 41 to 35 per 100,000 was not significant (*p* = 1.00). For violence and missing cases, significant reductions were observed in both sexes. Among men, violence-related TBI decreased by 64% and missing cases by 97% (both *p* < 0.0001). Among women, the corresponding reductions were 44% (*p* < 0.0001) and 99% (*p* < 0.0001), respectively. The reduction of the missing cases occurred mainly during 2008–2011. For the rest of the study period, from 2011 to 2022, this category contributed minimally to the total, between 0.4 and 3.2% for men and 0.1–2.5% for women, with a decreasing trend over time for both sexes.

### Fracture Rates by Sex and External Cause

Figure 1c and Table 2c show that fractures were more frequently caused by falls in women (53–61%) than in men (25–38%). Violence-related fractures were more common in men (17–28%) than in women (4–7%). A significant change over time was shown for women for fall-related fractures (*p* = 0.03) with a very slight increase, while no significant changes over time were observed for men (*p* = 0.34). Changes in transport-related fractures were significant for both sexes (*p* < 0.0001), although the change over time follows a stable trend from 2008 to 2022. Other significant changes over time included a 54% reduction in violence-related fractures among men and a 36% reduction among women (*p* < 0.0001 for both). Missing cases decreased by 98% (*p* = 0.0017) for men and 95% (*p* = 0.001) for women, mainly during the first years from 2008 to 2011. For the rest of the study period, from 2011 to 2022, this category contributed minimally to the total, between 0.1 and 0.6% for men and 0.1–0.4% for women, with a decreasing trend over time for both sexes. Additionally, other external causes were significant for both sexes, with a clear 35% reduction among men (*p* < 0.001) but a stable trend over time among women (*p* = 0.005).

### Age-Related Injury Patterns

Figure 2 illustrates mean head injury rates by age and external cause. Falls were the most common cause across all age groups, except among men aged 20–29, where violence occurred at a similar rate. Fall-related injuries showed a U-shaped age distribution, peaking in young children and the oldest-old. Transport- and violence-related injuries peaked during the teenage years and early adulthood. Among men aged 15–29, falls, transport, and violence all contributed to the peak, whereas among women, only falls and transport were dominant causes. Violence-related head injuries were common among men aged 15–29 but uncommon among women.

Figure 3 shows annual head injury rates by age group. Rates remained rather stable or declined in all groups under age 80. The 85+ group showed a significant increase by 52% for men and by 37% for women (both *p* < 0.0001). Among both men and women, the 80–84 age group also showed a significant 23% and 20% increase, respectively (*p* < 0.0001 and *p* = 0.002). Men aged 15–24 had higher rates and steeper declines than other young adult groups. Men aged 0–59 and 75–79 had significant changes over time (*p* = 0.001 for age 5–9 and *p* < 0.001 for the rest), while women had significant changes over time only for age 0–4 and 10–24 (*p* < 0.001).

### Injury Types for Traumatic Head Injuries

Figure 4 and Table 3 present annual incidence rate per 100,000 of specific injury types from 2008 to 2022. Concussion rates were significantly reduced in men (238–176 per 100,000, i.e., 26% reduction, *p* < 0.0001) and women (194–176, i.e., 10% reduction, *p* < 0.0001). Fracture rates decreased significantly by 25% in men (from 129 to 97 per 100,000, *p* < 0.0001). In women, strong evidence of a stable trend over time was found (*p* = 0.0007), with minimal change over time.

**Table 3.** Annual variation in proportions (denoted as mean, with range in parentheses) of specific traumatic head injury types, relative to all traumatic head injuries, 2008–2022

	Men	Women
A. Most common head injuries		
Concussion	55.6% (51.3–58.7%)	66.5% (61.5–69.4%)
Fractures of cranial and facial bones	27.8% (26.3–29.9%)	19.2% (17.8–21.1%)
B. Extra-axial haemorrhages		
EDH	0.5% (0.4–0.8%)	0.4% (0.3%–0.5%)
SDH	8.6% (6.0%–12.3%)	6.8% (4.9%–9.6%)
SAH	3.0% (1.4%–4.7%)	2.9% (1.4%–4.5%)
C. Other intracranial injuries		
Oedema	0.1% (0.1%–0.2%)	0.1% (0.1%–0.2%)
Diffuse	1.1% (0.5%–2.8%)	1.3% (0.4%–3.4%)
Focal	0.9% (0.7%–1.3%)	0.6% (0.4%–1.1%)
Other (aggregated)	2.2% (2.0%–3.1%)	2.1% (1.5%–3.2%)

SDH, subdural haemorrhage; SAH, subarachnoid haemorrhage; EDH, epidural haemorrhage.

Among extra-axial haemorrhages (shown in Fig. 4b), SDH and SAH showed sharp increases and highly significant changes over time. From 2008 to 2022, for both sexes, SDH nearly doubled in injury rate, while SAH approximately tripled in injury rate. SDH increased by 75% in men (from 24 to 42 per 100,000, *p* < 0.0001) and by 93% in women (from 14 to 27 per 100,000, *p* < 0.0001). SAH increased by 167% in men (from 6 to 16, *p* < 0.0001) and by 225% in women (from 4 to 13, *p* < 0.0001). SDH accounted for 6.0–12.3% of all head injuries and 68.7–72.9% of extra-axial haemorrhages in men, and 4.9–9.6% of all head injuries and 65.3–71.9% of extra-axial haemorrhages in women. SAH accounted for 1.4–4.7% of all head injuries and 17.4–27.3% of extra-axial haemorrhages in men and 1.4–4.5% and 21.0–32.0% in women, respectively. Epidural haemorrhage represented a minor portion of extra-axial haemorrhages, accounting for 0.4–0.8% in men and 0.3–0.5% in women, and decreased by 54% in men (*p* < 0.001) and 26% in women (*p* = 0.006).

Other intracranial injuries (shown in Fig. 4c) showed all highly significant decreases for both sexes (*p* < 0.0001 for all). These included oedema (64% reduction for men and 57% for women), diffuse TBI (87% reduction in both sexes), focal TBI (61% reduction for men and 62% in women), and aggregated “other” injuries (49% reduction in men and 46% in women).

## Discussion

This study presents nationwide epidemiological data on traumatic head injuries in Sweden, including all specialised care visits from 2008 to 2022. Unlike many previous studies, it reports injury rates from both inpatient and outpatient specialised care and separately for fractures and specific types of TBIs. Falls remained the leading external cause of head and brain injury, with their relative contribution increasing over time, while transport-related injuries accounted for 10–20% of cases. The results showed a general decline in head injury rates over the 15-year period. However, subdural and subarachnoid haemorrhages (SDH and SAH) doubled in incidence between 2008 and 2022.

Age-related differences were evident, with individuals aged 85+ showing a large and significant increase from 2008 to 2022, with approximately twice the incidence compared to other age groups. This increase is likely driven by the rising rates of SDH and SAH in both sexes. Head injury rates among women have not declined as much as in men, especially for fall- and transport-related injuries. For example, fracture and concussion rates decreased significantly in men but remained steady in women (shown in Fig. 4a), which likely explains the smaller overall reduction in head injury rates among women (shown in Fig. 1a). Our data show increasing head injury trends among older adults (either 65+ or 85+) consistent with previous studies [13–18], with falls as the main cause. Among these studies, Kannus and colleagues [17] covered the longest study period, from 1970 to 2017, followed by Hartholt and colleagues [13] from 1986 to 2010. Both studies reported increasing age-adjusted injury rates for age 80+ [17] and 65+ [13]. Kannus and colleagues [17] mentioned that the alarming rise has continued since their previous study [19]. Regarding traumatic haemorrhages, Hartholt and colleagues [13] and Peeters and colleagues [15] (including contusion as well) showed increasing trends for age 65+. Our longitudinal data, presented in both Figures 2 and 3, are consistent with these previous studies. The increasing magnitudes were generally comparable to that reported by Kannus and colleagues [17]. The increasing trend in our study extends the findings of our previous Swedish studies [11, 12]. The continued increase among the oldest-old, as well as increases in SDH and SAH, highlights the vulnerability of the ageing population. The question is if the worrying trends in age 85+ and SDH/SAH occurred due to increased exposure with a growing ageing population (more older adults at risk) or caused by increased vulnerability (substantially higher injury risk with age).

### Rising Incidence of SDH and SAH in the Older Adults Population

The increasing rates of SDH and SAH have occurred for more than 30 years, consistent with our earlier Swedish studies based on inpatient data from 1987 to 2010 [11, 12]. Because SDH and SAH are diagnosed with CT scans in specialised care, the injury rates reported in this study are most likely to approximate the true incidence. These injuries are strongly associated with ageing [20], as elderly individuals are more susceptible to bridging vein rupture due to larger cortical relative motion associated with brain atrophy [21].

Although multiple studies have reported traumatic head injury rates among older adults, broad, open-ended older age groups remain commonly used [22]. Age standardisation is used to account for potential age composition changes, and crude rates with too large age-specific groups can be misleading for longitudinal trend comparisons [23]. Our injury data for 85+ were aggregated, which prohibited us from presenting age-specific or age-adjusted trends in this study. Disaggregated data are needed to evaluate effects due to age composition changes over time and/or injury vulnerability, especially across oldest-old age groups. To uncover some of the uncertainty in the interpretation of our oldest-old injury data, we evaluated population data from Statistics Sweden (shown in Fig. 5). Both aggregated age 85+ and age composition were evaluated, using 5-year age groups from ages 85 to 99, and combined for 100–109 years old due to a small population. Even though we did not provide age-specific SDH/SAH trends, we do suspect that the aggregated incidence of SDH/SAH in Figure 4 is responsible for the alarming trends for the oldest-old, considering the age-related injury mechanism.

**Fig. 5.**
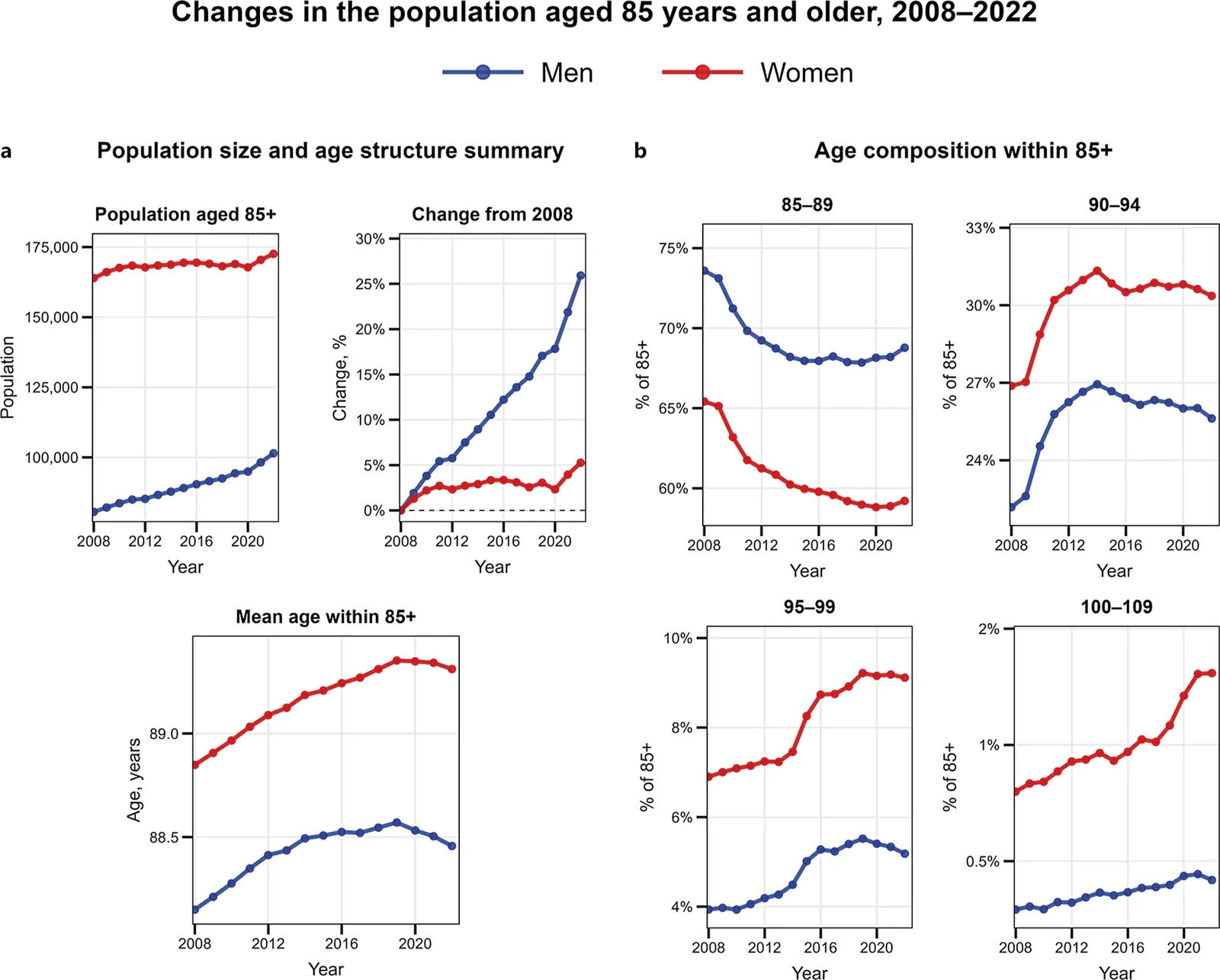
**a**, **b** Population size and age composition, for age 85+ (https://www.scb.se/hitta-statistik/sverige-i-siffror/manniskorna-i-sverige/sveriges-befolkning/) [24].

The population trends in Figure 5 show a marked increase in 85+ for men (up to 25% increase from 2008 to 2022) and a slight increase for women (up to 5% from 2008 to 2022). Women have a larger oldest-old population than men throughout the study period and around 75% larger in 2022 than 2008. The age composition plots show that the largest share is predominantly ages 85–89 with a reduction of around 5%, mainly during the beginning of the study period. The share for age 90–94 is three- to four-fold larger than age 95–99, increasing from 2008 to 2013 and were rather unchanged from 2016 onwards. Meanwhile, the 95–99 age group has the opposite trend with a larger increase in the middle of the study period but is also rather unchanged in the later years. Altogether, these trends indicate a demographic shift, where the age composition leans towards higher ages of the oldest-old during the second half of the study period.

The demographic shift displayed in the population data cannot alone explain the alarming trends for age 85+ in Figure 3 and SDH/SAH in Figure 4, due to a lack of age-specific injury data. However, the injury counts are directly proportional to injury rate and population, which likely suggests higher baseline vulnerability in the growing ageing population and more older adults at risk. For example, although men have higher SDH/SAH injury rates, the increase over time is larger for women (93% vs. 75% for SDH, 225% vs. 167% for SAH), which potentially is due to the higher share of ages 90+ for women, while men have a higher share than women for ages 85–89. As mentioned about the alarming trends from Kannus and colleagues [17], the authors mention that they could not rule out if the cause was an increase in falls, an increase in TBI after a fall, or both. Collectively, we believe our study present the need for future studies to present injury-specific data such as SDH/SAH, but with proper age-specific aggregation for age 85+. The injury-specific trends and age-disaggregated strata should also be evaluated to avoid violating data protection rights [22].

In summary, increasing trends of SDH and SAH in Sweden have been shown since 1997 [12], similar to the alarming TBI trends in Finland [17]. Our study continues to show that it is likely SDH and SAH specifically that concerns mainly older adults. The mechanisms of fall-related SDH and SAH among older adults need to be further studied. Specifically, the concerning trends are increasing with no sign of reduction, while targeted injury prevention initiatives are currently lacking.

### Current Initiatives to Reduce Fall-Related Injuries

Our findings that falls have surpassed RTAs as the primary cause of TBIs are consistent with earlier Swedish studies conducted more than 25 years ago [11, 12] and with the existing European reviews by Peeters and colleagues [2] and Brazinova and colleagues [4]. As steps beyond the current literature, we have included 15-year data from all specialised care visits, while also presenting injury rates stratified by causes for TBIs and fractures separately. This stratification is currently lacking in most injury epidemiology studies. Collectively, our study strengthens inferences from a comprehensive epidemiological view of traumatic head injuries, which is essential to inform effective countermeasures.

Transport-related head injuries have declined in Sweden, largely due to the implementation of Vision Zero. When introduced, Vision Zero initiated a shift to prioritise the understanding of human biomechanical tolerance and injury mechanisms to develop effective countermeasures. Although falls are the most common injury type in road traffic environments, they are not classified as transport-related injuries [25, 26]. To address this gap, Sweden became the first country to include serious pedestrian fall injuries in its Vision Zero targets, aiming for a 25% reduction by 2030 [27]. This goal is monitored based on data from the Swedish Traffic Accident Data Acquisition (STRADA), which integrates police and emergency hospital reports [28]. As of 2024, the Swedish Transport Administration reports no clear reduction in pedestrian falls [27], suggesting that current countermeasures may be insufficient. However, such conclusions may depend on the data quality of STRADA, which in turn relies on consistent hospital reporting. The last comparison between STRADA and NPR was conducted in 2012 [29], and an updated mapping would be needed to assess current estimates of fall-related head injuries among pedestrians. Recent studies show that low-energy falls among the elderly are increasing [17, 18, 30, 31]. Our data confirm rising head injury rates in the 85+ age group, mirroring trends in SDH and SAH. Although we did not stratify injury type by external cause, it is reasonable to assume that falls are the predominant cause of SDH and SAH in Sweden. The proportions of SDH and SAH resulting from pedestrian falls remain unknown and warrant further investigation.

Various fall prevention strategies exist, including physical therapy, road maintenance, and anti-slip footwear. However, the continued rise in severe TBIs, like SDH and SAH, suggests these interventions may be insufficient. The proportion of falls occurring in different environments is unclear, and better understanding of where pedestrian falls happen could guide more effective interventions. Also, greater insight into fall injury mechanisms is needed to reduce injury severity, as fall prevention alone cannot eliminate accidents and their consequences.

### The True Incidence of Concussion Rates Is Underestimated

Globally, concussion rates are estimated as 740 per 100,000 people [1], making it the most common form of TBI. Yet, many cases likely go untreated and are not captured in hospital data [32]. Cassidy and colleagues [6] found that hospital-reported rates (100–300 per 100,000) significantly underestimate true incidence, which may be closer to 600 per 100,000.

Regional studies support that concussion rates from inpatient care are underestimated, but the external validity of these studies is limited. Feigin and colleagues [7] identified 1,369 TBI cases in an urban and rural population in New Zealand, finding that 95% were mTBI, with 36% diagnosed outside hospital settings. No moderate-to-severe TBIs were diagnosed outside hospital settings. Skandsen and colleagues [33] analysed a cohort of individuals aged 16–59 in Trondheim, Norway, using data from both hospitals and general practitioners. They found the highest incidence of mTBI among teenagers, and notably, 71% of all cases did not require hospital admission. In Denmark, Eggertsen and colleagues [9] reported an increase in concussion rates from 280 to 308 per 100,000 between 1999 and 2018, based on all specialised care visits (inpatient and outpatient, but not including primary care). In our study, concussion rates in 2022 were 176 per 100,000 for both sexes – still likely underreported. Compared to the latest Swedish study by Pedersen and colleagues [12], who found a concussion rate of 90 per 100,000 using inpatient data from 2010, our 2010 rates (219 for men, 188 for women) demonstrate the impact of including outpatient care data. The initiative to reduce unnecessary CT scans, combined with fewer hospital beds and improved concussion treatment routines, may explain why concussions are now more often diagnosed outside hospitals or in outpatient care. While our study highlights the importance of comprehensive data to avoid underestimating concussion incidence, including primary care data would improve the estimates further. Although Sweden has prioritised access to primary care data with healthcare reforms [34], there is currently no nationally coordinated database that enables extraction of comprehensive primary care data, which limits national-level injury data analyses. NPR remains the most viable source, although the quality of outpatient data is still improving, which affects longitudinal studies of injury trends [35]. That said, the trends observed in the present study do not appear to be driven by reporting inconsistencies. The missing data were greatly reduced during the first years of the study period (2008–2011), mainly due to improved quality of outpatient care data, and remained minimal and stable for the rest of the study period.

## Limitations

This study included only primary diagnoses to focus on the main injury mechanisms. Including only primary diagnoses likely underestimates mTBI rates, as many concussions may be reported as secondary or tertiary diagnoses. Future studies should include all unique concussion diagnoses in specialised care. This could potentially improve estimates of concussion incidence, particularly given that national-level access to longitudinal primary care data is currently not possible.

Another limitation regarding the incidence estimation is the arbitrary 2-year washout period. Previous Swedish studies about sickness absence due to traumatic injuries, using NPR data, have utilised a washout period between 1 and 3 years [36, 37]. Our 2-year washout period reflects a compromise between excluding follow-up visits and retaining new incidences. Long-term complications may extend beyond 2 years, especially in elderly patients, and fall accidents occur quite often in this population. Hence, to determine a less arbitrary washout period, a sensitivity study is needed [38].

A major limitation in injury epidemiology studies is lack of disaggregation in data [22]. Our data only included crude injury rates for the aggregated age 85+ group, instead of 5-year age-specific data. Disaggregated age groups at 85+ would permit age-standardised data for the oldest-old to be presented. Even though some injuries might result in small numbers that can violate data protection rights, it is evident that SDH/SAH incidence should be presented in disaggregated age groups up to a reasonable upper age limit.

The population analyses in Figure 5 reveal some details about more older adults at risk due to increased population size and changes in age composition with a larger proportion of age 90+. However, we could not evaluate if increased vulnerability occurs with age in the oldest-old. Age-specific data for SDH/SAH could provide better understanding about age-related injury mechanisms.

The main priority of this study was to provide longitudinal injury rate trends from all specialised care visits, with a focus on providing key implications about current demands to improve traumatic head injury prevention. The importance of using statistical models to account for linear and nonlinear trends, compared to previous studies providing mainly descriptive statistics, was to properly evaluate the meaning of temporal trends. Emphasis was placed on the importance of identifying significant results for both steep changes such as for the oldest-old (alarming trends) and stable/unchanged patterns such as for fall-related TBIs (signalling either successful countermeasures or lack of injury prevention). As an injury epidemiological study, it was justified to ensure stringent control of false positives by reducing the risk of type I errors, achieving a balance between conservative testing and false discovery rates. The risk of false positives without obvious, or significant, trends can lead to implications that have no meaningful real-life application and should not be prioritised. Future data collection should provide possibilities of creating relevant strata (e.g., concussion-fall), as well as including longer study periods, to improve statistical power and strengthen any epidemiological inferences. Also, facilitating the lack of data resources, such as a nationwide primary care data system for traumatic injuries, is needed to improve estimates of high-incidence, mild injuries (e.g., concussion) due to the healthcare reforms.

### The Importance of Targeted Traumatic Head Injury Prevention

Our study identified reliable trends of increasing rates of SDH and SAH and increased head injury rates among the elderly, not only driven by age-related injury mechanisms but mainly by demographic shifts with a growing oldest-old population. Injury-specific knowledge is needed for developing effective fall countermeasures to accommodate for more older people at risk. While real-world fall monitoring is challenging, recent video-based studies in long-term care facilities have provided valuable insights [39]. For example, Komisar and colleagues [40] found that men had higher head injury risk despite women experiencing more fall-related head impacts and being more prone to fall accidents. This may help explain our findings of higher injury rates in men, particularly in older age groups and for SDH/SAH. Such data and insights into fall injury mechanisms are important for both preventing falls (e.g., understanding balance and fall strategies) and reducing the injury severity when falls are unavoidable (e.g., reduce impact loading). Biomechanical insights are essential for targeted injury prevention.

Our epidemiological data suggest increased vulnerability with age with more older adults at risk annually during the 15-year study period. It is important to consider not only the increased risk of falling but also the increased risk of injury per fall. While fall accident mechanisms are relatively well studied, more research is needed on injury-specific mechanisms to better interpret epidemiological trends. A Vision Zero framework for the prevention of fall-related injuries is urgently needed to address the vulnerability of the growing oldest-old population.

## Conclusion

Our study examined the incidence of traumatic head injuries in Sweden from 2008 to 2022. Unlike previous Swedish national studies that relied solely on inpatient data, we included both inpatient and outpatient specialised care records, improving estimates of head trauma – especially for injuries such as concussion. Our results showed an alarming increase in head trauma among the oldest-old, particularly in SDH and SAH, with falls being the leading injury mechanism. These findings have important implications for injury prevention. The disparate trends in incident rates across different accident and injury types underscore the need for targeted injury-specific prevention strategies rather than a one-size-fits-all approach. While Vision Zero has successfully driven research funding and interventions that reduced transport-related injuries, urgent actions and financial investments are clearly needed to address fall-related head injuries, particularly among the elderly.

## Acknowledgments

This work has been funded by the Swedish Foundation for Strategic Research (SSF). The authors are grateful to Joel Granwald and Henrik Nordin at the earliest stage, both at the National Board of Health and Welfare, for their help with acquiring the detailed datasets from the National Patient Register.

## Statement of Ethics

Ethical approval was not required for this study because the dataset obtained from the Swedish National Patient Register was fully anonymised before access. As the data are anonymised and individual identities are unknown to the researchers, informed consent could not be obtained.

## Conflict of Interest Statement

The authors declare no conflicts of interest.

## Funding Sources

The research has received funding from the Swedish Foundation for Strategic Research (SSF), no. FID22-0023.

## Author Contributions

Jia-Cheng Xu: conceptualisation, data curation, formal analysis, funding acquisition, investigation, methodology, project administration, validation, visualisation, and writing – original draft. Zhou Zhou: supervision and writing – review and editing. Christer Ahlström: supervision, validation, and writing – review and editing. Svein Kleiven: conceptualisation, resources, supervision, and writing – review and editing. All authors revised and approved the final manuscript, had full access to the dataset, and had final responsibility for the decision to submit for publication.

## Data Availability

The data of this study are not openly available but can be retrieved from the corresponding author upon reasonable request.
